# Occurrence, virulence genes, and antimicrobial profiles of *Escherichia coli* O157 isolated from ruminants slaughtered in Al Ain, United Arab Emirates

**DOI:** 10.1186/s12866-020-01899-0

**Published:** 2020-07-16

**Authors:** Dawood Al-Ajmi, Shafeeq Rahman, Sharmila Banu

**Affiliations:** Department of Integrative Agriculture, College of Food and Agriculture, United Arab Emirates University (UAEU), Al Ain, United Arab Emirates

**Keywords:** *Escherichia coli* O157, Antimicrobial resistance, Food pathogen testing

## Abstract

**Background:**

Shiga toxin-producing *Escherichia coli* (STEC) is a major source of food-borne illness around the world. *E. coli* O157 has been widely reported as the most common STEC serogroup and has emerged as an important enteric pathogen. Cattle, in particular have been identified as a major *E. coli* O157:H7 reservoir of human infections; however, the prevalence of this organism in camels, sheep, and goats is less understood. The aim of this study was to evaluate the occurrence and concentration of *E. coli* serotype O157 in the feces of healthy camels (*n* = 140), cattle (*n* = 137), sheep (*n* = 141) and goats (*n* = 150) slaughtered in United Arab Emirates (UAE) for meat consumption between September 2017 and August 2018. We used immunomagnetic separation coupled with a culture-plating method to detect *E. coli* O157. Non-sorbitol fermenting colonies were assessed via latex-agglutination testing, and positive cultures were analyzed by performing polymerase chain reactions to detect genes encoding attaching and effacing protein (*eaeA*), hemolysin A (*hlyA*, also known as *ehxA*) and Shiga toxin (*stx1* and s*tx2*), and *E. coli* O157:H7 specific genes (*rfb O157*, *uidA*, and *fliC*). All *E. coli* O157 isolates were analyzed for their susceptibility to 20 selected antimicrobials.

**Results:**

*E. coli* O157 was observed in camels, goats, and cattle fecal samples at abundances of 4.3, 2, and 1.46%, respectively, but it was undetectable in sheep feces. The most prevalent *E. coli* O157 gene in all STEC isolates was *stx*_*2*_;_*,*_ whereas, *stx*_1_ was not detected in any of the samples. The fecal samples from camels, goats, and cattle harbored *E. coli* O157 isolates that were 100% susceptible to cefotaxime, chloramphenicol, ciprofloxacin, norfloxacin, and polymyxin B.

**Conclusion:**

To our knowledge, this is the first report on the occurrence of *E. coli* O157 in slaughter animals in the UAE. Our results clearly demonstrate the presence of *E. coli* O157 in slaughtered animals, which could possibly contaminate meat products intended for human consumption.

## Background

Access to safe food is a basic human need but remains a major health concern across the world, with food-borne infections considered as a major global public health issue. These infections not only affect the health and well-being of consumers, but also adversily impact economies in food-exporting countries. Consequently, the frequency with which food items are rejected at border crossings due to microbial and chemical contaminants has increased dramatically over the past few years, resulting in huge economic losses and food wastage for both importing and exporting countries [1, 2]. Specifically, the lack of efficient food-safety programs in Arab countries prevented the export of food products into the international market in 2003 [3]. Raw meat, salads, and unpasteurized dairy products pose important food-contamination risks and increase the burden of food-borne diseases [4]. However, a few Arab countries have carried out microbial-contamination profiling regarding food-borne outbreaks [3]. As a result, 1926 cases of food-borne illness were reported in Lebanon in 2003 and 779 cases were reported in Libya in 2004, while 112,904 cases of acute gastroenteritis and diarrhea were reported in Oman in 2002 [3].

Recently, enterohemorrhagic strains of *Escherichia coli* have emerged as significant enteric pathogens. During the early 1980s, various serotypes were implicated in human disease, and *E. coli* O157 was the most prevalent. This serotype is responsible for severe abdominal illness, specifically enterohemorrhagic colitis (HC) and hemolytic uremic syndrome (HUS), and generally causes severe diarrhea [5, 6]. *E. coli* O157 belongs to the larger category of Shiga toxin-producing *E. coli* (STEC), which can produce Shiga toxin type 1 (*Stx*_*1*_), Shiga toxin type 2 (*Stx*_*2*_), or both, along with other variants [7]. *E. coli* O157 can infect humans via various routes*;* however, a large proportion of infections and human outbreaks have occurred following the consumption of contaminated food products, such as meat and dairy products [8]. Although the prevalence of STEC infections is often low, major outbreaks resulting in serious medical conditions have occurred throughout the world [9].

Typically, healthy cattle serve as the primary reservoir for STEC, but they are also carried by sheep and other animals [10]. Several host and farm practices have been associated with an increasing *E. coli* O157:H7 prevalence, including the animal age, season, and herd type [11, 12]. Currently, another major concern for human health is the rise in antimicrobial resistance due to the overuse of antibiotics in livestock production and human diseases treatment in developing countries [13, 14]. Various studies have been conducted worldwide to better understand the antimicrobial-resistance profiles of food-borne pathogens. However, no studies have been conducted to study the burden and drug-sensitivity profile of *E. coli* O157 in the United Arab Emirates (UAE). An abattoir or slaughterhouse is a place that has been approved by an authority to practice hygienic slaughtering and processing of meat products for human consumption [15]. Due to increases in the global population and urbanization, improper slaughterhouse practices can facilitate the growth and spread of pathogenic organisms if the slaughtered animals harbor pathogens in their feces. Thus, it is necessary to determine the prevalence and concentration of *E. coli* O157 in fecal samples in order to evaluate the extent of the risk for contamination. Such assessment would necessitate the development of cost-effective and safe interventions to control *E. coli* O157 shedding at the farm level to prevent further food-chain contamination.

Thus, this study aimed to evaluate the occurrence, concentration, virulence genes, and antimicrobial-resistance profiles of *E. coli* O157 in feces of healthy camels, cattle, sheep, and goats slaughtered for meat consumption. This is anticipated to be the first report of the occurrence and abundance of *E. coli* O157 in these animals within the UAE.

## Results

Examination of fecal samples collected from a slaughterhouse in Al Ain, UAE over ten months revealed that the occurrences of *E. coli* serotype O157 in camels, goats, and cattle were 4.3, 2, and 1.46%, respectively. However, *E. coli* O157 was not detected in the sheep fecal samples. Furthermore, no significant differences were observed in the occurrence of *E. coli* O157 between male and female animals or between different breeds. Given that no other groups have reported the prevalence of *E. coli* O157 in slaughtered animals in the UAE, we performed microbiological and molecular tests to confirm the presence of *E. coli* O157 strains.

Biochemical tests confirmed the presence of *E. coli* O157 strains. The strains were sorbitol and indole-negative, but positive for citrate utilization. Bacterial enumeration was carried out by plating the samples and manually counting the colonies to quantify the colony-forming units (CFUs). The CFU concentrations (CFU g^− 1^ feces) are presented in Table 1. The results indicate that the fecal concentrations of *E. coli* O157 were sufficiently high to promote the spread of infection. We also sought to understand the seasonal prevalence of *E. coli* O157 and found that during the ten-month study, *E. coli* O157 isolates were only obtained from the samples in February, March, and April. According to the National Center of Meteorology, the temperature range during these three months was 37 °C–45 °C. There was no statistically significant difference (*p* > 0.05) in the prevalence of *E. coli* O157 between sex, origin, or breed. However, due to the low number of positive samples and the prevalence, it was difficult to perform a comprehensive statistical analysis.

**Table 1:** *E. coli* O157-positive cases and the *E. coli* O157 concentrations in animal feces

Species	Number of animals	***E. coli*** O157-positive animals	Percentage (%)	***E. coli*** O157 Concentration (mean CFU per g feces)
Camel	140	6	4.3	4 × 10^4^
Goat	150	3	2.0	4 × 10^4^
Cattle	137	2	1.46	2 × 10^3^
Sheep	141	0	0.0	0

CFU colony-forming unit, used to estimate the number of viable bacteria cells in a sample

### Polymerase chain reaction (PCR) analysis

Out of the 12 positive camel fecal samples, five (42%) lacked the *fliC*H7 gene, and the virulence gene, *hlyA,* was present in nine samples (75%). In addition, the *eaeA* gene was present in 11 of the 12 camel fecal samples. Similarly, of the two positive cattle samples, one contained the *flic*H7 gene. Further, the *hlyA* gene was present in only one of the cattle samples, whereas the *eaeA* gene was present in both samples. All ten *E. coli* O157-positive goat samples (100%) lacked the *flic*H7 gene. Alternatively, the *hlyA* and *eaeA* genes were present in 6/10 (60%) of the goat samples. The most prevalent gene in the *E. coli* O157 isolates from all species was *stx*_*2*_, whereas *uid*A and *stx*_1_ was absent in all samples (Table 2). For some positive culture samples, we were unable to confirm the presence of *E. coli* O157 by PCR amplification, and those samples were considered negative for *E. coli* O157.

**Table 2:** Distribution of virulent genes detected by PCR

Sample number	Species	Genes						
		*rfbE*	*FlicH7*	*hlyA*	*uidA*	*eaeA*	*stx2*	*stx1*
1	Camel	+	+	–	–	+	+	–
2	Camel	+	+	+	–	+	+	–
3	Camel	+	–	–	–	+	+	–
4	Camel	+	+	–	–	–	+	–
5	Camel	+	+	+	–	+	+	–
6	Camel	+	–	+	–	+	+	–
7	Camel	+	–	+	–	+	+	–
8	Camel	+	+	+	–	+	+	–
9	Camel	+	+	+	–	+	+	–
10	Camel	+	+	+	–	+	+	–
11	Camel	+	–	+	–	+	+	–
12	Camel	+	–	+	–	+	+	–
13	Cattle	+	–	–	–	+	+	–
14	Cattle	+	+	+	–	+	+	–
15	Goat	+	–	–	–	–	+	–
16	Goat	+	–	+	–	+	+	–
17	Goat	+	–	+	–	+	+	–
18	Goat	+	–	–	–	–	+	–
19	Goat	+	–	–	–	–	+	–
20	Goat	+	–	+	–	+	+	–
21	Goat	+	–	+	–	+	+	–
22	Goat	+	–	+	–	+	+	–
23	Goat	+	–	+	–	+	+	–
24	Goat	+	–	–	–	–	+	–

### Antimicrobial susceptibility of isolates

All samples isolated from goats, cattle, and camels were subjected to antimicrobial susceptibility testing. We found that all (100%) of the *E. coli* O157 isolates were susceptible to cefotaxime, chloramphenicol, ciprofloxacin, norfloxacin, and polymyxin B. Kanamycin showed growth-inhibition zones with 20 isolates out of 24 positive samples. Other antimicrobials showed irregular susceptibility and resistance patterns that could not be considered effective (Table 3).

**Table 3:** Antimicrobials used, their symbols, and corresponding zones of inhibition for gram-negative enteric bacteria

Antimicrobial used	Symbol	Diameter of zone of inhibition (mm)		
		Resistant	Intermediate	Susceptible
Amoxicillin (25 μg)	AML	≤13	14–16	≥17
Ampicillin (10 μg)	AMP	≤13	14–17	≥18
Bacitracin (10 μg)	B	≤14	15–16	≥17
Cefotaxime (30 μg)	CTX	≤14	15–22	≥23
Cefoxitin (30 μg)	FOX	≤14	15–17	≥18
Ceftazidime (10 μg)	CAZ	≤12	13–17	≥18
Cefuroxime sodium (5 μg)	CXM	≤14	15–22	≥23
Chloramphenicol (30 μg)	CHL	≤14	15–20	≥21
Ciprofloxacin (30 μg)	CIP	≤15	16–20	≥21
Cloxacillin (5 μg)	CLOXA	≤13	14–16	≥17
Doxycycline (30 μg)	DO	≤13	14–16	≥17
Gentamycin (10 μg)	GEN	≤12	13–15	≥16
Kanamycin (30 μg)	K	≤13	14–17	≥18
Nalidixic acid (30 μg)	NAL	≤13	14–18	≥19
Nitrofurantoin (300 μg)	F	≤13	14–17	≥18
Norfloxacin (10 μg)	NOR	≤12	13–16	≥17
Polymyxin B (300 units)	PMB	≤8	9–11	≥12
Streptomycin (10 μg)	STR	≤11	12–14	≥15
Tetracycline (30 μg)	TET	≤12	15–18	≥19
Vancomycin (30 μg)	VAN	≤14	15–16	≥17

## Discussion

Human *E. coli* O157:H7 infections primarily originate from animal food sources [16]. Specifically, domestic ruminants, including cattle, sheep, and goats, have been reported to be major natural reservoirs for *E. coli* O157 and contribute significantly to the epidemiology of human infections [17]. In this study, we surveyed the occurrence and concentration of *E. coli* O157 (CFU g^− 1^ feces) in fecal samples from animals in a slaughterhouse in Al Ain, UAE. We successfully identified *E. coli* O157 in 4.3, 2, and 1.46% of the camel, goat and cattle samples, respectively, with concentrations ranging from TFTC (too few to count) to 4 × 10^4^ CFU g^− 1^ feces. This observed *E. coli* O157:H7 prevalence in the UAE slaughterhouse is in agreement with previous studies carried out in countries such as Ethiopia, South Africa, the United Kingdom, and Ireland, where the prevalences of *E. coli* O157:H7 in abattoirs were reported as 2.7, 2.8, 2.9 and 3.2% respectively [18–22]. Furthermore, in this study, the highest prevalence of *E. coli* O157 (4.3%) was found in camel feces. This result was consistent with the reports from neighboring countries, including Saudi Arabia (2.4%) [23], but slightly lower than those found in Ethiopia (8%) and Iran (6.4–9.6%) [24, 25]. Moreover, reports from Qatar showed that *E. coli* O157 was ten times more abundant in camel fecal samples than in cattle fecal samples [26]. Interestingly, Al-Gburi (2016) [27] reported that in Iraq, camels harbored the *E. coli* O157 most frequently (19%) among all animals tested, and these isolates were found to be multi-drug resistant.

Previously, researchers studying UAE camels were unable to isolate *E. coli* O157 from their fecal samples. A study conducted by Moore et al. [28] on racing camel calves from the UAE failed to show the presence of *E. coli* O157, and similar results were obtained by El-Sayed et al. [29] in a large herds of camels. However, this discrepancy with our results may be due to the absence of a reliable “gold standard” for detecting the presence of STEC *E.coli* O157 in samples, as well as the inherent difficulty associated with directly comparing the results obtained in this study with those obtained in other studies that used different culture methods. In addition, results from above studies were primarily based on the characterization of very few colonies picked from the samples, without specific screening and isolation techniques. In contrast, rather than using a direct culturing method, we employed enrichment in buffered-peptone water (BPW), followed by immunomagnetic separation of *E. coli* O157 (IMS) and culture on cefixime-tellurite sorbitol MacConkey (CT-SMAC) agar, to improve the isolation efficiency [30]. All isolates were confirmed as *E. coli* O157 via latex agglutination testing and PCR.

However, we were unable to identify *E. coli* O157 strains from sheep fecal samples. Similar results were published by Alhelfi et al. [31], where no *E. coli* O157 was detected in either cattle or sheep fecal samples within the target area studied in the United Kingdom. The failure to detect positive samples may be indicative of several factors, such as the study design, sample size, geographical origin, age and sex of the animals, abattoir conditions, animal husbandry, and/or the diet, which have been shown to impact the prevalence rates of *E. coli* O157 in livestock [32, 33]. In addition, a previous prevalence study [34] on suckling lambs did not detect *E. coli* O157 in all the 265 animals studied. However, it is worth noting that the samples in that study were not collected during the summer months, which might have affected the presence of *E. coli* O157 strains. The relationship between *E. coli* O157 and the gastrointestinal microbiome flora is not well understood. Indeed, factors encoded within the locus of enterocyte effacement (LEE), such as intimin, the translocated intimin receptor (*tir*), the *EspA* translocator, and other candidate bacterial factors have been implicated in the colonization and persistence of the strain in tested experimental ruminants such as sheep and cattle [35, 36]. It is important to note that in those previous experiments, the researchers screened neither for other pathogenic serogroups of *E. coli* that may be prevalent in sheep, nor for other bacterial pathogens. Thus, the absence of these strains in sheep does not indicate that sheep pose zero risk. Our study did not have access to sufficient information regarding the local sheep population, including animal ages, breeds, husbandry, and diet history. Hence, additional studies on larger herds, using a meta-analysis approach, are recommended to confirm the absence of *E. coli* O157 in local sheep population.

Although cattle were previously reported to be the most common *E. coli* O157 reservoir [37], in our study we only observed a prevalence of approximately 1.46%. Alternatively, data from a study carried out in Riyadh, Saudi Arabia, showed that 10.7% of cattle feces samples contained *E. coli* O157:H7. Moreover, cattle from Jordan had a higher prevalence of *E. coli* O157:H7 (12.22%) than cattle from other countries in Asia [38], and the lowest prevalence was reported in Taiwan (0.13%) [39]. In another report, it was shown that the prevalence rate of *E. coli* O157:H7 in fecal samples from cattle throughout the world ranged from 2.4 to 24% [40].

We also examined whether seasonal variation occurred in the prevalence of *E. coli* O157, as such fluctuations in cattle and meat products reportedly influence human *E. coli* O157 infection rates [41, 42]. Specifically, the prevalence in cattle feces are low in the winter and high in the spring, peaking in summer [43]. The warmer summer months may provide more suitable environments for *E. coli* O157:H7 survive and potentially multiply outside of the host in soil, feed, and water, resulting in a continual source of infection or re-infection of cattle populations. Al Ain, UAE, is located in a tropical dry area where the average temperature during different seasons can enhance bacterial growth. In fact, previous findings have shown that the prevalence of *E. coli* O157 increased during the summer months and declined in the winter [44]. Our results showed that the highest prevalence occurred in spring and the early summer months (February to April). This may likely reflect the adverse effect of the intense heat during the summer months in the UAE, when daytime temperatures may reach as high as 50 °C, causing *E. coli* 0157:H7 to decline due to the inability of the organism to persist in the extreme environment. However, our findings are in agreement with previous studies carried out in Riyadh, Saudi Arabia, where the highest prevalence was reported in the spring and early autumn months [23]. In fact, we were unable to detect *E. coli* O157:H7 in any other months, similar to what reported in other parts of the world.

The concentrations of *E. coli* O157 within the animal fecal samples were also quantified. In camels, the CFUs ranged from TFTC to 4 × 10^4^ CFU g^− 1^ feces, whereas the goat and cattle samples contained 4 × 10^4^ CFU g^− 1^ feces and 2 × 10^3^ CFU g^− 1^ feces, respectively. These results demonstrate that animals infected with *E. coli* O157 could be considered super shedders. Such high fecal *E. coli* O157 levels present the carcasses of these animals as a high risk for contamination if the proper precautions are not taken. Animals in Scottish slaughterhouses reportedly shed bacteria at a level of > 10^4^ CFU/g, constituting > 96% of the bacteria that were shed by all animals tested [45]. Together, these findings underscore the importance of the presence of high-shedding animals in a herd to the overall prevalence of colonization in the entire cattle population [45].

Shiga toxins are associated with HC and HUS, while intimin is responsible for attaching/effacing (A/E) lesions on intestinal epithelial cells. As such, enterohemolysins have been proposed as potential epidemiological markers for STEC strains [46]. Intimin, encoded by the *eaeA* gene, adheres to intestinal mucosa and causes intestinal lesion formation [47]. Enterohemolysin, encoded by *hly*A, leads to erythrocyte lysis, which may contribute to iron intake by intestinal bacteria [48]. Previous reports showed that *E. coli* O157:H7/H^−^ strains isolated from the feces of slaughtered ruminants exhibited *stx2* gene prominence over the *stx1* gene [34, 49, 50]. In our study, *stx*1 was absent from all strains irrespective of the species. Data from various studies have shown that *stx*2 and *eae*A are clinically important virulence genes, and the carriage of these genes was associated with the severity of human disease, especially HUS [51, 52]. Hence, our present data suggest that *E. coli* O157 strains harboring the major virulence genes may be more virulent to humans than strains lacking these genes. Nevertheless, it has been reported that the production of major virulence genes are not essential for pathogenesis, as several sporadic cases of HUS were induced by *Stx* and *eae*A-negative strains [53].

Antimicrobial resistance has been recognized as a global health issue for many decades. Food animals are considered key reservoirs of antibiotic-resistant bacteria because certain antibiotic-resistance genes identified in the bacteria of animal food products have also been identified in humans [54]. However, effectively treating *E. coli* O157:H7/H- infections is challenging, due to differing opinions presented by various investigators [55]. Many studies have reported an increasing incidence of multi-drug resistant *E. coli* O157:H7/H- strains isolated from the feces of slaughtered ruminants [56–58]. Our results show that all isolated strains were susceptible to antibiotics; cefotaxime, chloramphenicol, ciprofloxacin, norfloxacin, and polymyxin B. These findings are in agreement with previous reports of the antimicrobial-resistance patterns of *E. coli* O157:H7 isolates from animal and human sources [59, 60]. Further, previous reports on antibiotic-resistant bacteria in the feces of slaughtered ruminants demonstrated that the isolates showed high or intermediate resistance against multiple antimicrobials [56–58]. In fact, one study found that gentamycin resistance was the most common (56.0%), followed by ampicillin (48.0%), erythromycin (40.0%), amoxicillin (16.0%), tetracycline (12.0%), chloramphenicol (8.0%), nalidixic acid (8.0%), and streptomycin (4.0%) [57]. The high levels of resistance to the antibiotics evaluated in that study perhaps reflect their wide use in the veterinary sector for treating various infections. Antimicrobial resistance emerges due to widespread use of antibiotics in animals and humans, which subsequently transfers the resistance genes and bacteria between animals and products, humans, and the environment [61]. It is worth considering that antibiotic resistance in food-borne pathogens have increased virulence and can increase the burden on human health by increasing the risk of contracting an infection in immunocompromised persons and reducing the treatment options for illnesses. In this study, most isolates displayed an intermediate resistance profile, indicating the potential for resistance to occur in the future. Attention must also be given to the elevated intermediate susceptibility profiles of such antibiotics. Some of the drugs we studied such as amoxicillin and nalidixic acid are commonly prescribed for *E.coli* O157 treatment in adults but showed resistance in our study. There have been limited studies on antibiotic resistance in *E. coli* O157 strains isolated from ruminant feces in the UAE. However, testing of only a small study population for antibiotic susceptibility, variability in specific resistance genes within isolates, differences in preferred antibiotics, or origins of strains, may impact the observed resistance or even lead to false conclusion of higher resistance rates.

## Conclusions

In this study, the presence of the *E. coli* O157 serotype, it’s major virulence genes, and associated antibiotic resistance were investigated in strains isolated from the feces of slaughtered ruminants in the UAE, including, camels, cattle, sheep and goats. The presence of high concentrations of pathogens in some animals, along with seasonal variations, highlights the need for improved risk-mitigation strategies to screen for high-shedding animals prior to slaughter. In this study, we established the presence of high-shedding animals at slaughterhouses in the UAE, which pose increased contamination risks for both the food chain and the environment. Our results also demonstrate that bacterial shedding at a high concentration by a few animals may play a more significant role in pathogen dissemination than the prevalence rate, as it can directly cause carcass contamination. Although using antibiotics to treat *E. coli* O157 infections is controversial, monitoring antibiotic resistance in the strains continues to be useful for epidemiological purposes. Moreover, further studies are needed to accurately determine the level of *E. coli* O157 contamination in the carcasses and hides of animals in slaughterhouses. This study has some limitations specifically related to the small sample size. The number of positive samples was insufficient to detect monthly variations in the prevalence within statistically acceptable confidence limits; however, it did enable detection of the minimum prevalence rate in the species studied.

This work clearly supports the need for a platform aimed at designing regular screening programs in food safety and processing units, in order to ensure early and rapid detection of food-borne pathogens. The need for suitable control measures for such animals cannot be underestimated, and further studies are needed to devise mitigation strategies that will reduce the risk of large-scale contamination of the food chain or environment. Studies are also needed to characterize the contamination of *E. coli* O157 isolates and other non-*E. coli* O157 pathogens in meat food products.

## Methods

### Animal fecal samples

This study was conducted on healthy camels,cattle, sheep and goats slaughtered in the public abattoir in Al Ain, UAE, during the study period. Most animals were transported to the slaughterhouse and, hence, we studied the animals starting from the lairage. Samples were collected for a period of ten months, from September 2017 to July 2018. As per the project design, the samples were collected and tested on a biweekly basis, and the breed, species, gender, and fecal consistency of the animal were noted. All animals were local, except for the cattle. The mature Holstein dairy cattle were culled from a local dairy farm. A total of 568 fecal samples were collected from healthy animals in the slaughterhouse (sheep: 141, goats: 150, camels: 140, cattle: 137). Approximately 10 cm of the recto-anal junction was excised immediately after the slaughter and the fecal samples were collected, stored on ice, transported to our laboratory, and examined. Microbial testing was performed within 3 h of sample collection.

### IMS technique

Approximately 10 g of each fecal sample was diluted in 90 mL of BPW (Oxoid) and mixed for 30 s. A 15 mL aliquot of each fecal mixture was stored for later use. Next, 1 mL of enriched fecal sample was mixed with 20 μL of magnetic beads coated with an anti-O157 antibody and IMS was performed according to the manufacturer’s instructions (Oxoid). The bead suspension (100 μL) was then plated onto CT-SMAC agar (Oxoid). The plates were incubated at 37 °C for 24 h and presumptive *E. coli* O157 colonies were identified as *E. coli* O157 that did not ferment sorbitol. These colonies were picked and inoculated into nutrient agar slants at 37 °C for 24 h and stored in a refrigerator for further biochemical analysis. These isolates were further verified using conventional biochemical tests as described by Harrigan [62]. Tests were performed to examine indole production and motility using Sulfide Indole Motility (SIM) medium (Merck); citrate utilization with Simmons citrate agar (Merck); and methyl red and vogues-Proskauer using MR-VP medium (Merck) [63].

### Latex agglutination test

*E. coli* O157 strains were confirmed by growing non-sorbitol-fermenting colonies (white-gray color) on CT-SMAC medium and agglutinating then with *E. coli* O157 latex reagent (Oxoid), as per the manufacturer’s instructions. Isolates showing visible agglutination following reaction with the test latex solution were again subcultured for virulence gene identification.

### Enumeration of *E. coli* O157

Latex agglutination test-positive samples were directly enumerated. Briefly, 100 μL of each enriched fecal sample was serially diluted three times in 900 μL of BPW (i.e., diluted by 10^1^-, 10^2^-, and 10^3^-fold). The serially diluted samples were separated by IMS and the number of *E. coli* O157 cells was counted [64]. Finally, 100 μL of separated bacteria–bead complexes were plated onto two SMAC plates (50 μL/plate) and incubated at 37 °C for 18–24 h. After incubation, the CFUs were counted and recorded. The CFUs ranged from too few to count (TFTC) to too numerous to count (TNTC).

### DNA extraction

After enumerating the positive samples from each animal in each collection, they were subjected to DNA extraction, as previously described [65]. Pure culture samples were centrifuged at 10,000 rpm for 1 min, and each supernatant was discarded and re-suspended with sterile water. The tubes were incubated at 100 °C in a dry bath for 10 min, after which they were transferred to ice for 5–10 min. Crude DNA in the supernatant was obtained by centrifugation. The DNA was later analyzed for the presence of virulence genes.

### Confirmation of *E. coli *O157 by multiplex PCR

All biochemically confirmed *E. coli* isolates that were O157 agglutination-positive were tested for the presence of genes in the *E. coli* O157 antigen gene locus, *rfbE*O157:H7, which codes for GDP perosamine synthetase (*rfbO157*), *uidA*, and the H7 flagellar protein (*fliC*H7) by multiplex PCR analysis, as previously described by Al-Ajmi et al. [65]. Briefly, 1 μL of crude DNA extract was amplified in 25 μL mixtures containing 10 μL PCR master mix (Thermo Scientific), 5 μL nuclease free water, and 1 μL of each forward and reverse primer (Table 4) for *rfbE*, *Flic*H7, and *uidA.* A negative control without template DNA was also included in the analysis. The thermal cycling program was as follows: initial denaturation at 95 °C for 2 min; 30 cycles of denaturation (30 s, 94 °C), annealing (15 s, 58 °C), and extension (1 min 68 °C); and a final extension (10 min, 68 °C).

**Table 4:** Primers used to confirm the presence of *E. coli* O157:H7 and virulence genes

Target gene	Primer name	Primer sequence (5′–3′)	Amplification size (bp)
*rfbE*	O157AF	AAGATTGCGCTGAAGCCTTTG	497
	O157AR	CATTGGCATCGTGTGGACAG	
*fliC*	FLICH7 F	GCGCTGTCGAGTTCTATCGAGC	625
	FLICH7 R	CAACGGTGACTTTATCGCCATTCC	
*uid*A	PT-2	GCGAAAACTGTGGAATTGGG	252
	PT-3	TGATGCTCCATAACTTCCTG	
*hly*A	hlyA F	GCATCATCAAGCGTACGTTCC	534
	hlyA R	AATGAGCCAAGCTGGTTAAGCT	
*stx*2	Stx2F	GGCACTGTCTGAAACTGCTCC	255
	Stx2R	TCGCCAGTTATCTGACATTCTG	
*stx*1	Stx1F	ATAAATCGCCATTCGTTGACTAC	180
	Stx1R	AGAACGCCCACTGAGATCATC	

An additional multiplex PCR analysis was carried out using the *eae*A, *hly*A, *Stx*1, and *Stx*2 primers to quantify virulence genes (Table 4) [66]. Briefly, 1 μL of crude DNA extract was amplified in 20-μL reaction mixtures containing 10 μL of PCR master mix, 5 μL nuclease-free water, and 1 μL of each forward and reverse primer. The following thermal cycling program was used: 1 min at 94 °C; 30 cycles of 30 s at 94 °C, 1 min at 55 °C, 1 min at 72 °C and a 5 min extension at 72 °C, with a no-template negative control. The PCR products were separated on 2% agarose gels containing ethidium bromide, and DNA bands were visualized on an ultraviolet light box using a gel-image capture system and software (Bio-Rad).

### Antimicrobial testing

The Kirby–Bauer disk-diffusion method was performed to test nine antimicrobials, which are commonly used for treating human infections. An inoculum from each *E. coli* O157 strain was grown in 5 mL Mueller–Hinton (MH) broth (Himedia) at 37 °C and assessed for turbidity to a McFarland 0.5 standard, according to the Clinical and Laboratory Standards Institute (CLSI) [67] standards. All test discs were obtained from Oxoid. A standard reference strain (*E. coli* ATCC 25922), which is sensitive to all tested antimicrobial agents, was used as the control strain. MH agar plates were swabbed with sterile cotton swabs, and antimicrobial paper disks containing amoxicillin (25 μg), ampicillin (10 μg), bacitracin (10 μg), cefotaxime (30 μg), cefoxitin (30 μg), ceftazidime (10 μg), cefuroxime sodium (5 μg), chloramphenicol (30 μg), ciprofloxacin (30 μg), cloxacillin (5 μg), doxycycline (30 μg), gentamycin (10 μg), kanamycin (30 μg), nalidixic acid (30 μg), nitrofurantoin (300 μg), norfloxacin (10 μg), polymyxin B (300 U), streptomycin (10 μg), tetracycline (30 μg), and vancomycin (30 μg) were impregnated onto the surface of inoculated MH agar plates. The plates were incubated at 37 °C for 16–18 h, and the diameters of each zone of microbial-growth inhibition around the antimicrobial disk were measured. The minimum inhibitory concentration was determined for each antimicrobial agent, and each agent was characterized as being sensitive, having intermediate resistance, or being resistant according to criteria established by the CLSI [67].

### Statistical analysis

Statistical analysis of the breed of animal, sex, season of sampling and antimicrobial susceptibility were analyzed using the SAS software (SAS, V8.02) and comparisons between means were made using least significant difference at a *p* value of 0.05.

## Data Availability

The datasets used and/or analyzed during this study are available from the corresponding author upon reasonable request.

## Abbrevations

AML: Amoxicillin
AMP: Ampicillin
B: Bacitracin
BPW: Buffered Peptone Water
CAZ: Ceftazidime
CFUs: colony-forming units
CHL: Chloramphenicol
CIP: Ciprofloxacin
CLOXA: Cloxacillin
CLSI: Clinical and Laboratory Standards Institute
CT-SMAC: cefixime-tellurite sorbitol MacConkey
CTX: Cefotaxime
CXM: Cefuroxime sodium
DO: Doxycycline
F: Nitrofurantoin
FOX: Cefoxitin
GEN: Gentamycin
HC: enterohemorrhagic colitis
HUS: hemolytic uremic syndrome
IMS: Immuno Magnetic Separation
K: Kanamycin
MIC: Minimum Inhibitory Concentration
NAL: Nalidixic acid
NOR: Norfloxacin
PCR: Polymerase Chain Reaction
PMB: Polymyxin B
STEC: Shiga toxin-producing *Escherichia coli*
STR: Streptomycin
TET: Tetracycline
VAN: Vancomycin
